# Severity of hypoxic ischemic encephalopathy and heart rate variability in neonates: a systematic review

**DOI:** 10.1186/s12887-019-1603-7

**Published:** 2019-07-19

**Authors:** Mads Andersen, Ted C. K. Andelius, Mette V. Pedersen, Kasper J. Kyng, Tine B. Henriksen

**Affiliations:** Department of Pediatrics, Aarhus University Hospital, Palle Juul-Jensens Boulevard 99, 8200 Aarhus, Denmark

**Keywords:** Neonatology, Hypoxic ischemic encephalopathy, Neonatal encephalopathy, Heart rate variability

## Abstract

**Background:**

Several studies have investigated heart rate variability (HRV) as a biomarker for acute brain injury in hypoxic ischemic encephalopathy (HIE). However, the current evidence is heterogeneous and needs further reviewing to direct future studies. We aimed to systematically review whether HIE severity is associated with HRV.

**Methods:**

This systematic review was conducted according to the preferred reporting items for systematic review and meta analyses (PRISMA). We included studies comparing neonates with severe or moderate HIE with neonates with mild or no HIE with respect to different HRV measures within 7 days of birth. Article selection and quality assessment was independently performed by two reviewers. Risk of bias and strength of evidence was evaluated by the Newcastle-Ottawa scale (NOS) and the Grading of Recommendations Assessment, Development and Evaluation (GRADE).

**Results:**

We screened 1187 studies. From these, four observational studies with 248 neonates were included. For all HRV measures, the strength of evidence was very low. Neonates with severe or moderate HIE showed a reduction in most HRV measures compared to neonates with mild or no HIE with a greater reduction in those with severe HIE.

**Conclusions:**

Moderate and severe HIE was associated with a reduction in most HRV measures. Accordingly, HRV is a potential biomarker for HIE severity during the first week of life. However, the uncertainty calls for more studies.

**Electronic supplementary material:**

The online version of this article (10.1186/s12887-019-1603-7) contains supplementary material, which is available to authorized users.

## Introduction

The incidence of hypoxic ischemic encephalopathy (HIE) is estimated to be 1.5 per 1000 live births [1]. In survivors, HIE may result in brain damage with neurodevelopmental delay, cerebral palsy, or epilepsy [2–7].

HIE is graded (i.e., mild, moderate or severe) by clinical examination (Sarnat staging or Thompson score) [8, 9]. The clinical HIE grade combined with other measures, e.g., electroencephalogram (EEG) and neuroimaging, are used for estimating the severity and the risk of adverse neurodevelopmental outcome [10–13]. These assessments are critical for clinical decision making and for counselling parents. However, the prognostic value of these measures may be modest and they may be difficult to access, implement, or interpret [14–16].

Heart rate variability (HRV) describes the variation of time intervals between each subsequent heartbeat. HRV is controlled by the autonomic nervous system, which may be affected by hypoxia and compromised blood flow preceding HIE [8, 17]. HRV has the potential to become an objective, non-invasive, easily accessible, and continuous point-of-care measure. Therefore, it may be a valuable supplement to the current methods for initial HIE assessment, monitoring, selection of treatment, and prognosis. However, the clinical use is currently limited by a lack of standardized data in neonates and technical difficulties when analyzing HRV from electrocardiogram (ECG) at the bedside. Currently, the only clinical use of HRV is heart rate characteristics (HRC) obtained from HeRO® monitors, which analyze bedside ECG in order to predict onset of sepsis in neonates with very low birth weight [18]. Several studies have found a correlation between HIE and various HRV measures, suggesting that HRV is a potential biomarker for the severity of neonatal HIE [19–26]. However, the current evidence is heterogeneous and needs further reviewing to direct future studies.

The aim of this study was to systematically review whether the severity of HIE is associated with measures of HRV during the first week of life. Together with the recent review by Oliveira et al. [27], which focuses on HRV and its ability to predict brain injury and neurodevelopment outcome at ≥1 year of age, we feel that a number of aspects related to both the immediate association between HRV and HIE severity and the correlation to later outcomes have been thoroughly reviewed for the benefit of future studies.

## Methods

This review was conducted according to the preferred reporting items for systematic reviews and meta-analyses (PRISMA) [28]. Methods, search strategy, and inclusion criteria were pre-specified in a protocol registered with the International Prospective Register of Systematic Reviews (PROSPERO) and published on 14 April 2018 (registration number: CRD42018090638) [29].

### Study selection and eligibility criteria

#### Types of studies

Studies written in English and published in journals with peer review were included. No date restriction was implemented. Randomized controlled trials and observational studies were eligible for inclusion but case reports were excluded.

#### Types of participants

A study was eligible if the population contained neonates assessed for HIE within 24 h after birth.

#### Types of exposure and comparators

The exposure was defined as a clinical course, biochemical measures, and a neurological examination in keeping with HIE, followed by a classification of severe or moderate HIE by EEG, amplitude EEG, Sarnat staging, or Thompson Score. The comparators were defined as mild or no HIE.

#### Terminology

Neonatal encephalopathy may have specific etiologies other than hypoxia-ischemia. HIE implies that hypoxia-ischemia is known to have led to the clinical state [30]. However, we opted to continue to use the term HIE because the included studies used this terminology even though it may be unclear whether hypoxia-ischemia solely contributed to the encephalopathy.

#### Types of outcome measures

The outcome was defined as HRV measures assessed at the time of, or after, the HIE assessment and within 7 days after birth. The primary measure considered was the standard deviation of normal-to-normal intervals (SDNN) as it is more accessible and easier to interpret than most other HRV measures [31]. NN-intervals are defined as the intervals between adjacent QRS-complexes resulting from sinus node depolarizations [31]. Furthermore, we included other time domain measures, frequency domain measures, and non-linear measures. Other time domain measures included the standard deviation of RR-intervals (SDRR) and triangular interpolation of the NN-interval (TINN). SDRR contains intervals between each adjacent QRS-complex and therefore, as opposed to SDNN, also includes abnormal complexes. TINN is defined as the baseline width of the histogram showing the measured NN-intervals [31]. Frequency domain measures estimate the absolute or relative power within different frequency bands and includes very low frequency (VLF), low frequency (LF), high frequency (HF), and LF/HF ratio [31]. Non-linear measures, usually presented by Poincaré plots, describe the variation in different time-series of measures and includes Poincaré plot standard deviation perpendicular to the line of identity (SD1), Poincaré plot standard deviation following the line of identity (SD2), and the SD1/SD2 ratio [32].

### Search strategy

The specific search strategy for each database was developed in consultation with two medical librarians. Pubmed, Embase, Web of Science, Cochrane Database (CENTRAL), and Scopus were searched using subject headings and free text related to “*hypoxic ischemic encephalopathy*” and “*heart rate variability*”. The search was performed 6 April 2018. References of included studies were manually investigated. For PubMed and CENTRAL, the following search strategy was used: hypoxia ischaemia; hypoxic ischemia; hypoxic ischaemia; hypoxic ischemic; hypoxic ischaemic; HIE; asphyxia*; encephalopathy; asphyxia neonatorum[MeSH]; hypoxia-ischemia, brain[MeSH]. These were combined with: RR interval*; NN interval*; heart rate variation; HRV; time domain measure*; non-linear measure*; frequency domain measure*; heart rate variability; beat-to-beat variability; heart rate[MeSH]. The following Embase subject headings were: *newborn hypoxia/exp; hypoxic ischemic encephalopathy/exp; heart rate variability/exp.* For Scopus and Web of Science only free text was applied. The search results were then combined using Endnote X8.2® and Covidence systematic review software and duplicates were removed [33]. The complete search strategy is available in Additional file 1.

### Screening and data extraction

**Fig. 1:**
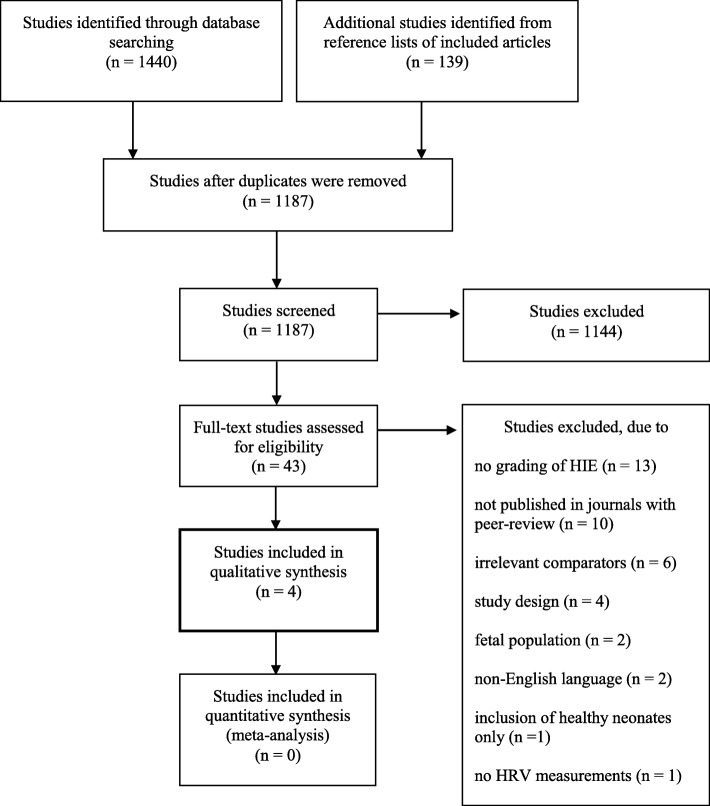
PRISMA flowchart of the study selection process

Two reviewers (MA and TCKA) independently screened titles and abstracts of the identified studies. Studies that met the eligibility criteria or provided insufficient information were subjected to full-text review. Assessment of potential eligibility and the subsequent data extraction was also performed by two reviewers (MA and TCKA). Any disagreements were resolved by discussion until consensus was reached or by consulting a third reviewer (TBH). The screening and selection process was documented in a PRISMA flowchart (Fig. 1) [28]. Data was extracted using a predefined data collection form based on the Cochrane Consumers and Communication Review Group Data Extraction Template [34]. To reduce errors and to identify missing data fields, piloting of the data collection form was performed by two reviewers (MA and TCKA). We did not contact authors of studies with missing or inadequate information.

The following information was extracted from the included studies [1]: title, author, country, year of publication, reference list, funding sources, and possible conflicts of interests [2]; type of methods including study design and unit of allocation [3]; characteristics of the neonates (gestational age, postnatal age, birth weight, and pathologies) including the eligibility criteria [4]; methods for assessing the HIE severity [5]; methods and postnatal age when assessing the HRV measures [6]; results and statistical significance [7]; information critical for providing an assessment of risk of bias and strength of evidence by the Newcastle-Ottawa Scale and the Grading of Recommendations Assessment, Development and Evaluation (GRADE).

### Risk of bias in individual studies

The risk of bias assessment was independently performed by two reviewers (MA and TCKA). Any disagreements were resolved by discussion until consensus was reached or by consulting a third reviewer (TBH). To assess the risk of bias in the included studies, a modified version of the Newcastle-Ottawa Scale for cohort studies was used as proposed by the Cochrane Handbook [34, 35]. Using this scale, studies were awarded points with a maximum of nine. The points were awarded based on three domains [1]: *selection*, scored by the representativeness of the neonates with severe or moderate HIE, selection of the neonates with mild or no HIE, ascertainment of the HIE severity, and demonstration that outcome was not present at study start [2]; *comparability*, scored by the comparability of cohorts based on design or analysis in the studies with regards to malformations, infections, metabolic diseases, gender, birth weight, gestational age, postnatal age, therapeutic hypothermia, and medication [3]; *outcome*, scored by the assessment of the HRV measurements, sufficient length of follow-up, and adequacy of follow-up of the neonates. A maximum of four, two, and three points could be awarded in each domain, respectively. The quality of the studies was rated as ‘good’, ‘fair’, or ‘poor’ according to the points awarded. ‘Good quality’ was given 3–4 points in selection and 1–2 points in comparability and 2–3 points in outcome; ‘fair quality’ was given 2 points in selection and 1–2 points in comparability and 2–3 points in outcome; and ‘poor quality’ was given 0–1 points in selection or 0 points in comparability or 0–1 points in outcome.

### Synthesis of results

Unless otherwise stated, the summary measure was expressed as mean difference with 95% confidence interval (CI). Statistical significance refers to a two-sided *p*-value of less than 0.05. Calculations were performed in *Review Manager 5.3* [36]. The heterogeneity between studies did not allow for meta-analysis. Hence, a narrative synthesis of the results was performed. The synthesis was made in accordance with Popay et al. [37]. We arranged our results based on the different combinations of comparisons between the neonates with severe or moderate HIE and the neonates with mild or no HIE. For each of these comparisons we present the results both tabulated and narratively summarised. Furthermore, we sought relations between each of these comparisons such as dose-response. We used the GRADE approach for assessing the strength of evidence [38]. The number of included studies was insufficient to allow construction for a funnel plot and formal testing of asymmetry. Selective reporting bias was assessed by comparing the outcomes reported in the method section and the result section of the included studies.

## Results

### Study selection

A total of 1440 studies were identified across the different databases. After removal of duplicates, 1048 studies were screened by title and abstract. A total of 43 full-text studies were assessed for eligibility. Of these, four studies met the inclusion criteria and were submitted to data extraction and analysis [19–22]. Furthermore, 139 studies from the reference lists of the included studies were screened but none of these were included (Fig. 1). The included studies were published between 2012 and 2017. An overview of excluded articles with reasons for exclusion is available in Additional file 2.

### Study characteristics

#### Setting, design, and number of participants

All the studies were conducted in a hospital setting and the neonates were recruited from neonatal intensive care units (NICUs). All studies were cohort studies. Data was available for a total of 248 neonates included in the four studies. Of these, 135 neonates provided 308 epochs with simultaneous ECG and EEG recordings. Number of neonates by study are provided in Table 1.

**Table 1:** Gestational age, birth weight, and male to female ratio of the neonates in the four studies on hypoxic ischemic encephalopathy (HIE) and heart rate variability

	HIE severity	Number of neonates	Gestational age (weeks)	Birth weight (g)	M/F-ratio
*Aliefendioglu* et al. *2012, Turkey* [19]	Severe HIE Moderate HIE	10 12	39 (0.78) ^*a*^ 39 (0.72) ^*a*^	3500 (515) ^*a*^ 3433 (557) ^*a*^	6/4 8/4
	No HIE	24	39 (0.88) ^*a*^	3283 (379) ^*a*^	14/10
*Vergales* et al. *2013, USA* [20]	Severe, moderate, mild, and no HIE	67	38 (1.4) ^*a*^	3236 (511) ^*a*^	NDA
*Goulding* et al. *2015, Ireland* [21]	Severe, moderate, and mild HIE	44	39 (36, 42) ^*b*^	3384 (1830, 5040) ^*b*^	26/18
	No HIE	17	40 (38, 41) ^*b*^	3601 (2980, 4060) ^*b*^	11/6
*Goulding* et al. *2017, Ireland* [22]	Severe, moderate, and mild HIE	74	40 (39, 41) ^*c*^	3425 (3165, 3745) ^*c*^	40/34

^a^ Values are presented as mean (standard deviation). ^b^ Values are presented as mean (minimum to maximum)

^c^ Values are presented as median (lower to upper quartile)

M/F-ratio, male to female ratio; NDA, no data available

#### Populations

Gestational age, birth weight, and male to female ratio by study can be seen in Table 1.

Aliefendioglu et al. [19] defined HIE as the presence of profound metabolic or mixed acidemia with pH < 7.00 in an arterial blood sample taken within 60 min after birth, an Apgar score of < 3 for longer than 5 min, abnormal neurology (seizures, coma, hypotonia), and multi-organ involvement. Neonates with either moderate or severe HIE was compared with healthy term controls who were matched by gender, gestational age, and postnatal age. Neonates with other pathologies including infectious diseases, congenital malformations, congenital heart diseases, and small for gestational age were excluded. How the neonates were sampled and during which period was not stated.

Vergales et al. [20] reviewed all records of infants with a clinical diagnosis of severe or moderate HIE from 2005 to 2011 admitted to a single NICU for therapeutic hypothermia. These were later classified by EEG and categorized into those with mild, moderate, or severe encephalopathy and those who were considered normal. Criteria for therapeutic hypothermia included gestational age ≥ 36 weeks, evidence of an acute perinatal hypoxic-ischemic event, and severe or moderate encephalopathy based on Sarnat staging [8, 39]. Neonates with a gestational age < 36 weeks were considered for cooling, and therefore inclusion in the study, on an individual basis.

Goulding et al. 2015 [21] included all term neonates with HIE from 2003 to 2007 admitted to two NICUs prior to the introduction of therapeutic hypothermia. These were later classified by EEG and divided into mild, moderate or severe HIE. The eligibility criteria were defined as the fulfilment of two or more of the following criteria: initial arterial pH < 7.1, Apgar score ≤ 6 at 5 min after birth, initial capillary or arterial lactate level > 7 mmol/l, or abnormal neurological examination or clinical seizures. A reference group of healthy neonates were sampled during the same study period, but how they were sampled was not stated.

Goulding et al. 2017 [22] included all term neonates with HIE from 2009 to 2014 admitted to a single NICU for therapeutic hypothermia. The HIE eligibility criteria were identical with Goulding et al. 2015 [21].

#### Classification of HIE severity

Only the study by Aliefendioglu et al. [19] classified HIE clinically according to the Sarnat staging [8]. Only neonates with moderate or severe HIE was considered exposed. In the remaining three studies, the severity of HIE was classified by multichannel video-EEG. Vergales et al. [20] classified EEG tracings based on Shellhaas et al. [40] which included evaluation of continuity/discontinuity, amplitude, symmetry, synchrony, lability of biobehavioral state, and composition of the EEG background. The EEG tracings were interpreted by a neurophysiologist. Goulding et al. [21] [22] classified the EEG tracings according to Murray et al. [12] who evaluated background amplitude, presence of discontinuity, duration of EEG activity burst and interburst interval, time prior to return of sleep-wake cycling, and presence of seizures. The EEG tracings were interpreted by two clinical physiologists.

#### Assessment of HRV

Aliefendioglu et al. [19] included mean LF, mean HF, and LF/HF ratio which were recorded at the end of the first postnatal week by ECG. The recordings were analyzed with spectral technique using Fast Fourier transform by a blinded pediatric cardiologist. The measurements were provided as relative power of the frequency bands in normal units (nu). The following frequency bands were applied: LF: 0.03–0.15 Hz and HF: 0.15–0.5 Hz.

Vergales et al. [20] obtained SDRR measurements from within the first 24 h of birth and until 10 days after birth. SDRR for each neonate was calculated as the standard deviation of sets of 4096 RR-intervals acquired from a heart rate characteristic index monitor.

Goulding et al. [21, 22] aimed at getting concurrent ECG and EEG recordings for 1 h at 12, 24, and 48 h and 6, 12, 24, 36, 48, and 72 h after birth, respectively. Each ECG recording was divided into 5 min’ segments and the HRV measures were estimated from the NN-intervals within each of these. The HRV measures included SDNN, TINN, mean VLF, mean LF, mean HF, and LF/HF ratio. From each neonate, the mean of the HRV measurements across all obtainable epochs were used in the statistical analysis and analysed in relation to the EEG tracings at each time point. The frequency domain measures were estimated as absolute power of the frequency bands (ms^2^) using a Periodogram [31]. The following frequency bands were applied: VLF: 0.01–0.04 Hz; LF: 0.04–0.2 Hz; HF: 0.2–2 Hz.

### Risk of bias within studies

The points awarded to each study by the Newcastle-Ottawa Scale are summarized in Table 2. The representativeness of the neonates with severe or moderate HIE was appropriate and the comparators was drawn from the same hospitals. The assessment of both HIE severity and HRV measures were done by examining the appropriate recordings. Only one study stated a blinded assessment of the HRV measures [19]. This study was also the only study reporting adjustment for potential confounders such as gestational age, birth weight, gender, and different pathological conditions. Vergales et al. [20] reported adjustment for administration of phenobarbital, while Goulding et al. 2015 [21] reported adjustments for both phenobarbital and morphine. These adjustments failed to change the estimated associations apart from the association between TINN and LF and the HIE severity which became statistically insignificant. Within all four studies, the neonates were comparable with respect to postnatal age and therapeutic hypothermia.

**Table 2:** Points awarded by the Newcastle-Ottawa Scale to the four studies on hypoxic ischemic encephalopathy (HIE) and heart rate variability (HRV)

	Selection				Comparability	Outcome			Quality score^i^
	Representativeness of exposed cohort^a^	Selection of non-exposed cohort^b^	Ascertainment of exposure^c^	Presences of outcome of interest^d^	Comparability of cohorts^e^	Assessment of outcome^f^	Long enough follow-up^g^	Adequacy of follow up^h^	
Aliefendioglu et al. 2012, Turkey [19]	A (✹)	A (✹)	A (✹)	A (✹)	A, B (✹✹)	A (✹)	A (✹)	A (✹)	Good quality
*Vergales* et al. *2013, USA* [20]	A (✹)	A (✹)	A (✹)	A (✹)	B (✹)	B (✹)	A (✹)	C	Good quality
*Goulding* et al. *2015, Ireland* [21]	A (✹)	A (✹)	A (✹)	A (✹)	B (✹)	B (✹)	A (✹)	A (✹)	Good quality
*Goulding* et al. *2017*, *Ireland* [22]	A (✹)	A (✹)	A (✹)	A (✹)	B (✹)	B (✹)	A (✹)	A (✹)	Good quality

^a^ A, truly representative; B, somewhat representative; C, selected group; D, no description of the derivation of the cohort

^b^ A, drawn from the community as the exposed cohort; B, drawn from a different source; C, no description of the derivation of the non-exposed cohort

^c^ A, secure record (e.g., surgical records); B, structured interview; C, written self-report; D, no description

^d^ Demonstration that outcome of interest was not present at the start of the study: A, yes; B, no.

^e^ Comparability of cohorts based of the design or analysis: A, study controls for the most important factor (malformation); B, study controls for any additional factor (infections, metabolic diseases, gender, birth weight, gestational age, postnatal age, therapeutic hypothermia, and medication)

^f^ A, independent blind assessment; B, record linkage; C, self-report; D, no description

^g^ Was follow-up long enough for outcomes to occur? A, yes; B, no.

^h^ A, complete follow-up - all subjects were accounted for; B, subjects lost to follow-up were unlikely to introduce bias - small numbers were lost (< 5%) or description was provided of those lost; C, follow-up rate < 95%, and there was no description of those lost; D, no statement

^I^ ‘Good quality’ was given 3–4 points (✹) in selection and 1–2 points in comparability and 2–3 points in outcome; ‘fair quality’ was given 2 points in selection and 1–2 points in comparability and 2–3 points in outcome; and ‘poor quality’ was given 0–1 points in selection or 0 points in comparability or 0–1 points in outcome

### Results of individual studies

The studies were heterogeneous which made direct comparisons between the studies problematic. Number of neonates or epochs in each statistical analysis and mean differences with 95% CI are reported in Table 3. Five different comparisons and ten different HRV measures were presented in the studies. Non-linear measurements, usually presented by Poincaré plots, were not reported.

**Table 3:** Mean differences (MD) in heart rate variability (HRV) between the different severities of neonatal hypoxic ischemic encephalopathy (HIE) by study

Severe HIE compared with mild HIE								
	Number in the statistical analysis	Time of assessment	TH	HRV measures	Mean, 95% CI (severe)	Mean, 95% CI (mild)	MD, 95% CI	*P*-values
*Goulding* et al. *2015, Ireland* [21]	Severe, *n* = 17 Mild, *n* = 40	12–48 h	No	SDNN (ms) TINN (ms) Mean VLF (ms^2^) Mean LF (ms^2^) Mean HF (ms^2^) LF/HF ratio	8.5 (5.1, 14) 24.5 (14.8, 40.6) 27.5 (9.7, 78.2) 13.2 (4.1, 42.8) 4 (2.4, 5.8) 3.7 (2.4, 5.9)	18.6 (15.4, 22.4) 50 (40.6, 61.6) 138.8 (95.3, 202.1) 84.1 (54.4, 130.2) 16.8 (10, 28.2) 5.6 (4.4, 7.2)	−10.1 (− 15.8, − 4.4) − 25.5 (− 42.2, − 8.8) − 111.3 (− 175.6, − 47.0) − 70.9 (− 113.4, − 28.4) − 12.8 (− 24.1, − 1.6) − 1.9 (− 4.1, 0.3)	< 0.01 < 0.01 < 0.01 < 0.01 0.03 0.09
*Goulding* et al. *2017*, *Ireland* [22]	Severe, *n* = 60 Mild, *n* = 110	6–72 h	Yes	SDNN (ms) TINN (ms) Mean VLF (ms^2^) Mean LF (ms^2^) Mean HF (ms^2^) LF/HF ratio	10.1 (7.6, 13.4) 26.8 (20.5, 35) 40.1 (21.7, 74.3) 18.1 (9.3, 35.2) 5.8 (3.2, 10.7) 3.3 (2.3, 4.7)	20.4 (17.2, 24.2) 51.9 (43.9, 61.6) 162.1 (112.8, 232.5) 92.1 (61.9, 137) 17.5 (12, 25.4) 5.6 (4.5, 6.9)	−10.3 (− 14.8, − 5.8) − 25.1 (− 36.6, − 13.6) − 122.0 (− 187.4, − 56.6) − 74.0 (− 113.7, − 34.3) − 11.7 (− 19.4, − 4.0) − 2.3 (− 4, − 0.6)	< 0.01 < 0.01 < 0.01 < 0.01 < 0.01 < 0.01
Moderate HIE compared with mild HIE								
	Number in the statistical analysis	Time of assessment	TH	HRV measures	Mean, 95% CI (moderate)	Mean, 95% CI (mild)	MD, 95% CI	P-values
*Goulding* et al. *2015*, *Ireland* [21]	Moderate, *n* = 16 Mild, n = 40	12–48 h	No	SDNN (ms) TINN (ms) Mean VLF (ms^2^) Mean LF (ms^2^) Mean HF (ms^2^) LF/HF ratio	11.2 (7.8, 16.1) 28 (18.8, 41.6) 57 (28.7, 113) 29.4 (12.6, 68.5) 3.6 (1.3, 7.6) 9.4 (6.3, 13.9)	18.6 (15.4, 22.4) 50 (40.6, 61.6) 138.8 (95.3, 202.1) 84.1 (54.4, 130.2) 16.8 (10, 28.2) 5.6 (4.4, 7.2)	−7.4 (− 12.8, − 2.0) − 22.0 (− 37.5, − 6.5) − 81.8 (− 149.8, − 13.8) − 54.7 (− 101.8, − 7.6) − 13.2 (− 23.2, − 3.2) 3.7 (− 0.3, 7.9)	< 0.01 < 0.01 0.02 0.03 0.01 0.08
*Goulding* et al. *2017*, *Ireland* [22]	Moderate, *n* = 48 Mild, *n* = 110	6–72 h	Yes	SDNN (ms) TINN (ms) Mean VLF (ms^2^) Mean LF (ms^2^) Mean HF (ms^2^) LF/HF ratio	15.1 (11.2, 20.3) 42.5 (31.8, 56.8) 90.9 (48.5, 170.4) 52.8 (9.3, 107.7) 16.8 (8.7, 32.8) 3.5 (2.3, 5.1)	20.4 (17.2, 24.2) 51.9 (43.9, 61.6) 162.1 (112.8, 232.5) 92.1 (61.9, 137) 17.5 (12, 25.4) 5.6 (4.5, 6.9)	−5.3 (− 11, 0.4) − 9.4 (− 24.7, 5.9) − 71.2 (− 156.6, 14.2) − 39.3 (− 101.2, 22.6) − 0.7 (− 14.5, 13.1) − 2.1 (− 3.9, − 0.3)	0.08 0.23 0.10 0.21 0.92 0.02
Severe HIE compared with no HIE								
	Number in the statistical analysis	Time of assessment	TH	HRV measures	Mean, 95% CI (severe)	Mean, 95% CI (No HIE)	MD, 95% CI	P-values
*Aliefendioglu* et al. *2012, Turkey* [19]	Severe, *n* = 10 No HIE, *n* = 24	End of first postnatal week	No	Mean LF (nu) Mean HF (nu) LF/HF ratio	27.8 (19.7, 35.9) 48.9 (44.5, 53.5) 0.6 (0.4, 0.9)	73.1 (70.3, 75.9) 20.3 (18.1, 22.5) 4 (3.3, 4.7)	−45.3 (− 53.8, − 36.8) 28.6 (23.7, 33.5) − 3.4 (− 4.1, − 2.7)	< 0.01 < 0.01 < 0.01
*Goulding* et al. *2015, Ireland* [21]	Severe, n = 17 No HIE, *n* = 17	12–48 h	No	SDNN (ms) TINN (ms) Mean VLF (ms^2^) Mean LF (ms^2^) Mean HF (ms^2^) LF/HF ratio	8.5 (5.1, 14) 24.5 (14.8, 40.6) 27.5 (9.7, 78.2) 13.2 (4.1, 42.8) 4 (2.4, 5.8) 3.7 (2.4, 5.9)	32.9 (27.9, 38.8) 106.4 (93.5, 119.3) 422.1 (296.8, 598.2) 375.7 (263.5, 535.6) 93.6 (61.7, 142) 4.4 (3.3, 5.9)	−24.4 (−31.5, − 17.4) − 81.9 (− 100.1, − 63.6) − 394.6 (− 548.7, − 240.6) − 362.4 (− 499.9, − 225.0) − 89.7 (− 130.4, − 49.0) − 0.7 (− 2.9, 1.5)	< 0.01 < 0.01 < 0.01 < 0.01 < 0.01 0.54
Moderate HIE compared with no HIE								
	Number in the statistical analysis	Time of assessment	TH	HRV measures	Mean, 95% CI (moderate)	Mean, 95% CI (No HIE)	MD, 95% CI	*P*-values
*Aliefendioglu* et al. *2012, Turkey* [19]	Moderate, *n* = 12 No HIE, *n* = 24	End of first postnatal week	No	Mean LF (nu) Mean HF (nu) LF/HF ratio	57.8 (52.5, 63.1) 30.6 (26.6, 34.6) 1.9 (1.5, 2.4)	73.1 (70.3, 75.9) 20.3 (18.1, 22.5) 4 (3.3, 4.7)	−15.3 (− 21.3, − 9.3) 10.3 (5.8, 14.8) − 2.1 (− 2.9, − 1.3)	< 0.01 < 0.01 < 0.01
*Goulding* et al. *2015, Ireland* [21]	Moderate, n = 16 No HIE, n = 17	12–48 h	No	SDNN (ms) TINN (ms) Mean VLF (ms^2^) Mean LF (ms^2^) Mean HF (ms^2^) LF/HF ratio	11.2 (7.8, 16.1) 28 (18.8, 41.6) 57 (28.7, 113) 29.4 (12.6, 68.5) 3.6 (1.3, 7.6) 9.4 (6.3, 13.9)	32.9 (27.9, 38.8) 106.4 (93.5, 119.3) 422.1 (296.8, 598.2) 375.7 (263.5, 535.6) 93.6 (61.7, 142) 4.4 (3.3, 5.9)	−21.7 (−28.6, − 14.8) − 78.4 (− 95.6, − 61.2) − 365.1 (− 521.1, − 209.1) − 346.3 (− 485.2, − 207.4) − 90.1 (− 130.4, − 49.7) 4.9 (0.9, 9.0)	< 0.01 < 0.01 < 0.01 < 0.01 < 0.01 0.02
Severe or moderate HIE compared with mild or no HIE								
	Number in the statistical analysis	Time of assessment	TH	HRV measures	Mean, 95% CI (severe/moderate)	Mean, 95% CI (mild/no HIE)	MD, 95% Cl	P-values
*Vergales* et al. *2013, USA* [20]	Severe/moderate, *n* = 25 Mild/no HIE, n = 11	Within first 24 h	Yes	SDRR (ms)	17.6 (13.8, 21.4)	26.8 (20.1, 33.5)	−9.2 (− 16.5, − 1.9)	0.02
	NDA	1–7 days	Yes	SDRR (ms)	NDA	NDA	NDA	< 0.05 at day 1 and from day 3–7

*TH* therapeutic hypothermia, *CI* confidence interval, *SDNN* standard deviation of normal-to-normal intervals, *TINN* triangular interpolation of normal-to-normal intervals, *VLF* very low frequency, *LF* low frequency, *HF* high frequency, nu, normal units, *SDRR* standard deviation of RR-intervals, *NDA* no data available

#### Severe HIE compared with mild HIE

Goulding et al. [21, 22] found a statistically significant reduction in all HRV measures in neonates with severe HIE compared with neonates with mild HIE with exception of the LF/HF ratio in Goulding et al. 2015 [21] (Table 3).

#### Moderate HIE compared with mild HIE

Goulding et al. 2015 [21] found a statistically significant reduction in all HRV measures in neonates with moderate HIE compared with neonates with mild HIE with exception of the LF/HF ratio. However, Goulding et al. 2017 [22] found no association with exception of a reduced LF/HF ratio in neonates with moderate HIE (Table 3).

#### Severe HIE compared with no HIE

Aliefendioglu et al. [19] found a statistically significant mean difference in frequency domain measures when comparing neonates with severe HIE with neonates with no HIE. Severe HIE was associated with reduced LF and increased HF with a subsequent reduced LF/HF ratio (Table 3). In contrast, Goulding et al. [21, 22] found a reduced HF in neonates with severe or moderate HIE. However, these studies measured the absolute power of the frequency bands while Aliefendioglu et al. [19] measured the relative power. Goulding et al. 2015 [21] found a statistically significant reduction in all other HRV measures in neonates with severe HIE compared with neonates without HIE with exception of the LF/HF ratio (Table 3).

#### Moderate HIE compared with no HIE

Aliefendioglu et al. [19] found a statistically significant mean difference in frequency domain measures when neonates with moderate HIE were compared with neonates without HIE. Thus, the same tendency was identified as when severe HIE was considered. However, the mean differences in LF and HF were significantly smaller (Table 3). Goulding et al. 2015 [21] found a statistically significant reduction in all HRV measures in neonates with moderate HIE compared with neonates without HIE with exception of the LF/HF ratio (Table 3).

#### Severe or moderate HIE compared with mild or no HIE

Vergales et al. [20] found a statistically significant reduction in SDRR within the first 24 h after birth when comparing neonates with severe or moderate HIE with neonates with mild or no HIE (Table 3). A figure presenting the results from day one to seven after birth was available, and the differences in SDRR was statistically significant at day one and from day three to seven. However, no evidence of association was found on day two. The mean differences and the precise number of neonates in the statistical analysis was not presented.

### Risk of bias across studies

We found no indication of selective reporting bias as no discrepancies between outcomes reported in methods and results were found in the included studies. The results included in this review are from relatively small studies and all found an association between the severity of HIE and HRV. Therefore, we were unable to rule out publication bias. This may lead to an overestimation of the association between HIE severity and HRV.

## Discussion

### Summary of evidence

One of the main findings in this systematic review was the lack of evidence in this specific area of interest especially due to the variety of HRV measures and methodology for measuring. For every outcome, the strength of the evidence was very low according to the GRADE assessment mainly due to the risk of bias and imprecision of estimates. Still, our review provides three key findings that may be of importance [1]: in three out of four studies, neonates with severe or moderate HIE had reduced time domain measures compared with neonates with mild or no HIE [2]; in three out of four studies, neonates with severe or moderate HIE had reduced frequency domain measures compared with neonates with mild or no HIE with exception of an increased HF in relative power [3]; in three out of four studies, a dose-response like pattern was seen as a tendency towards HRV being more reduced with increasing HIE severity.

Currently, no standardized methodology for measuring HRV in neonates exist. This is needed as the methods used may affect HRV measures. Time domain measures are affected by the sampling length of the ECG [31, 32]. For example, this issue hampered the comparison of HRV measures from Vergales et al. [20] and Goulding et al. [21] [22]. Frequency domain measures may be affected by differences in methodology (i.e., parametric or non-parametric) and the width of the frequency bands used [41]. Doyle et al. [42] has defined the frequency bands in healthy neonates less than 12 h of age as: VLF: 0.01–0.04 Hz; LF: 0.04–0.2 Hz; HF: > 0.2 Hz. These frequency bands were used by Goulding et al. [21, 22] but not by Aliefendioglu et al. [19], which again limit comparisons and meta-analyses. Therefore, we emphasize the need for standard guidelines for future studies on HRV in neonates. In the future, such guidelines would allow for collaborations and centre-to-centre data comparison.

Time domain measures are more accessible and easier to interpret compared with most other HRV measures [31]. SDRR was only reported in a single study comparing neonates with moderate and severe HIE with neonates with mild or no HIE determined by EEG after an initial clinical diagnosis of moderate or severe HIE [20]. However, only discrimination of the various grades of HIE will provide detailed information on HRV measures in HIE. This study also reported HRC monitor data. They found that the HRC index had an inverse correlation to SDRR and could predict adverse outcomes in neonates with HIE, indicating a possible clinical use of HRV in the assessment of HIE. SDNN describes the total variability and is believed to be controlled by both the sympathetic- and parasympathetic nervous system [31, 43]. Thus, SDNN may be the most clinically relevant HRV biomarker for HIE severity. Overall, neonates with severe or moderate HIE had a reduced SDNN compared with neonates with mild or no HIE. One study [22] found no statistically significant reduction in SDNN when neonates with moderate HIE were compared with neonates with mild HIE. The main difference between this study and the previous study from the same group [21] was treatment with therapeutic hypothermia in the later. However, other studies have found no effect of temperature on SDNN in neonates with HIE but have reported an increase in HF in absolute power during rewarming and a negative association between LF in relative power and temperature [26, 44].

None of the studies reported HRV measures from the first 6 h after birth. Assessment of the grade of HIE in this period is essential when considering therapeutic hypothermia since this treatment must be initiated as early as possible and preferably before 6 h after birth to be neuroprotective [45, 46]. Yamaguchi et al. [24] reported an increased SDNN within 0 to 6 h after delivery in preterm fetal sheep with severe hypoxia-ischemia compared with healthy controls or mild hypoxia-ischemia. In the secondary phase of brain injury, considered to be 6 to 72 h after delivery, SDNN decreased and was significantly reduced compared with the controls. These findings are in keeping with the findings of the clinical studies included in this systematic review.

Frequency domain measures also appeared to be potential markers of HIE severity. LF and HF may be used to describe the autonomic regulation of HRV but the interpretation of the individual frequency measures is still uncertain. LF may reflect a combination of parasympathetic activity, sympathetic activity, and baroreceptor regulation, or baroreceptor regulation only, while it is believed that HF mainly reflects the parasympathetic activity [31, 47–53]. Neonates with severe or moderate HIE had reduced frequency domain measurements in absolute power. The absolute power may reflect the overall autonomic activity, which therefore appears to be reduced [47]. Furthermore, neonates with severe or moderate HIE had a reduced LF/HF ratio in relative power. LF and HF in relative power reflects the proportion of the total power minus VLF, which may accentuate the balance between the different neuronal regulations of the HRV [31]. As HF is believed only to reflect the parasympathetic activity and LF may reflect different neural components, these findings may indicate a preponderance of parasympathetic activity in these neonates [19].

In frequency domain measures in relative power, a dose-response-like relationship was indicated by a larger difference when comparing neonates with severe HIE with no HIE than when comparing neonates with moderate HIE with no HIE. The same pattern was seen in time domain measures and most frequency domain measures in absolute power, although not statistically significant. Still, these findings indicate that HRV may be a potential biomarker for HIE severity. This is supported by other studies who found that the HRV could distinguish neurodevelopment outcome in neonates with HIE. Goulding et al. 2015 [21] also investigated two-year neurodevelopmental outcome and found a correlation between adverse outcomes and reduced HRV measurements from 12 to 48 h after birth. Massaro et al. [25] compared neonates with HIE with either favorable or adverse outcomes at 15 months. Frequency domain measures were measured in relative power and assessed between 5 to 110 h after birth. In neonates with an adverse outcome, LF was reduced while HF was increased, which corroborated with the findings in Aliefendioglu et al. [19] HRV seemed to provide the best discrimination between those with adverse outcome and those without if measured at 24 to 80 h of life. A systematic review [27] has evaluated HRV as a long-term prognostic marker. They concluded that HRV may be used as a long-term prognostic marker for neurodevelopmental outcome in HIE. However, further research was warranted.

### Limitations

#### Strength

This systematic review followed a comprehensive and structured search strategy implemented in five different databases. The study followed an a priori registered protocol, minimizing the risk of reporting bias. Furthermore, screening, selection of studies, data extraction, assessment of risk of bias, and strength of evidence was carried out independently by two reviewers. Reporting bias was not identified. However, the possibility of publication bias could not be ruled out.

#### Limitations

Meta-analysis was impossible due to heterogeneity between studies with respect to the assessment of both HIE and HRV. Many of the comparisons presented in this review were not controlled for important variables such as gestational age, birth weight, and different pathological conditions e.g., malformations and sepsis [54–57]. It is well known that these factors may depress HRV measures and thereby influence the results [54–57]. Thus, confounding from these variables may influence the results. Even when adjusting for these different variables, there is a potential risk of residual confounding. Furthermore, the studies offered no description of the course of delivery. Therefore, neonates with encephalopathy caused by other etiologies than hypoxia-ischemia may have been included. Thus, it is possible that the identified HRV patterns reflects other etiologies of neonatal encephalopathy in addition to hypoxia-ischemia.

## Conclusion

Overall, this review found that moderate and severe HIE was associated with reduced HRV measures except from an increased HF in relative power. This indicates that HRV may have potential as a biomarker for HIE severity during the acute phase of injury. However, the strength of the evidence for each HRV measure was very low, and therefore further research is required. We recommend that future studies focus on [1]: larger sample sizes of well-defined populations of neonates [2]; a stringent definition of HIE grade [3] well described HRV measures with postnatal timing accurately recorded [4]; comparability between the neonates with different severities of HIE. This could minimize the risk of bias and imprecision, which we found to influence previous studies.

## Additional files

Additional file 1: The full search strategy. The full search strategies used in Pubmed, Embase, Web of Science, Cochrane Database (CENTRAL), and Scopus. (DOCX 17 kb)

Additional file 2: Excluded studies with reasons for exclusion. List of studies excluded with reasons for exclusion during the full-text assessment of eligibility. (DOCX 112 kb)

## Data Availability

All data generated or analyzed during this study are included in this published article and its supplementary information files.

## Abbreviations

ECG: Electrocardiogram
EEG: Electroencephalogram
HF: High frequency
HIE: Hypoxic ischemic encephalopathy
HRC: Heart rate characteristics
HRV: Heart rate variability
LF: Low frequency
NICU: Neonatal intensive care units
SD1: Poincaré plot standard deviation perpendicular to the line of identity
SD2: Poincaré plot standard deviation following the line of identity
SDNN: Standard deviation of NN-intervals
SDRR: Standard deviation of RR-intervals
TINN: Triangular interpolation of the NN-interval
VLF: Very low frequency
