# Supplementary material for: Severity of hypoxic ischemic encephalopathy and heart rate variability in neonates: a systematic review
Source: BMC Pediatr. 2019 Jul 19;19:242. doi: 10.1186/s12887-019-1603-7 (PMC6639904; doi:10.1186/s12887-019-1603-7)
Supplement: Supplementary file 1 — The full search strategy. The full search strategies used in Pubmed, Embase, Web of Science, Cochrane Database (CENTRAL), and Scopus. (DOCX 17 kb) [file 12887_2019_1603_MOESM1_ESM.docx]

**Additional file 1**

**Hypoxic ischemic encephalopathy and heart rate variability in neonates: a systematic review**

*Mads Andersen, Ted C. K. Andelius, Mette V. Pedersen, Kasper J. Kyng, Tine B. Henriksen*

**The full search strategy**

**Pubmed and The Cochrane database (CENTRAL)**

1. "hypoxia ischaemia"
2. "hypoxic ischemia"
3. "hypoxic ischaemia"
4. "hypoxic ischemic"
5. "hypoxic ischaemic"
6. "HIE"
7. "asphyxia*"
8. "encephalopathy"
9. "asphyxia neonatorum"[Mesh]
10. "Hypoxia-Ischemia, Brain"[Mesh]
11. "RR interval*"
12. "NN interval*"
13. "heart rate variation"
14. "HRV"
15. "time domain measure*"
16. "non-linear measure*"
17. "frequency domain measure*"
18. "heart rate variability"
19. "Heart rate"[Mesh]
20. “Beat-to-beat variability”
21. #1 OR #2 OR #3 OR #4 OR #5 OR #6 OR #7 OR #8 OR #9 OR #10
22. #11 OR #12 OR #13 OR #14 OR #15 OR #16 OR #17 OR #18 OR #19 OR #20
23. #21 AND #22

**Embase**

1. "hypoxia ischaemia"
2. "hypoxic ischemia"
3. "hypoxic ischaemia"
4. "hypoxic ischemic"
5. "hypoxic ischaemic"
6. "HIE"
7. "asphyxia*"
8. "encephalopathy"
9. "newborn hypoxia"/exp
10. "hypoxic ischemic encephalopathy"/exp
11. "RR interval*"
12. "NN interval*"
13. "heart rate variation"
14. "HRV"
15. "time domain measure*"
16. "non-linear measure*"
17. "frequency domain measure*"
18. "heart rate variability"/exp
19. "Heart rate variability"
20. “Beat-to-beat variability”
21. #1 OR #2 OR #3 OR #4 OR #5 OR #6 OR #7 OR #8 OR #9 OR #10
22. #11 OR #12 OR #13 OR #14 OR #15 OR #16 OR #17 OR #18 OR #19 OR #20
23. #21 AND #22

**Web of Science and Scopus**

1. "hypoxia ischaemia"
2. "hypoxic ischemia"
3. "hypoxic ischaemia"
4. "hypoxic ischemic"
5. "hypoxic ischaemic"
6. "HIE"
7. "asphyxia*"
8. "encephalopathy"
9. "hypoxic ischemic encephalopathy"
10. "RR interval*"
11. "NN interval*"
12. "heart rate variation"
13. "HRV"
14. "time domain measure*"
15. "non-linear measure*"
16. "frequency domain measure*"
17. "heart rate variability"
18. “beat-to-beat variability”
19. #1 OR #2 OR #3 OR #4 OR #5 OR #6 OR #7 OR #8 OR #9
20. #10 OR #11 OR #12 OR #13 OR #14 OR #15 OR #16 OR #17 OR #18
21. #19 AND #20
