# Supplementary material for: Severity of hypoxic ischemic encephalopathy and heart rate variability in neonates: a systematic review
Source: BMC Pediatr. 2019 Jul 19;19:242. doi: 10.1186/s12887-019-1603-7 (PMC6639904; doi:10.1186/s12887-019-1603-7)
Supplement: Supplementary file 2 — Excluded studies with reasons for exclusion. List of studies excluded with reasons for exclusion during the full-text assessment of eligibility. (DOCX 112 kb) [file 12887_2019_1603_MOESM2_ESM.docx]

**Additional file 2**

**Hypoxic ischemic encephalopathy and heart rate variability in neonates: a systematic review**

*Mads Andersen, Ted C. K. Andelius, Mette V. Pedersen, Kasper J. Kyng, Tine B. Henriksen*

**Excluded studies with reasons for exclusion**

| **Title** | **Authors** | **Main reason for exclusion** |
| --- | --- | --- |
| *Perinatal asphyxia, causes, detection and neurologic sequelae* | Adamsons, K.; Myers, R. E. | Study design |
| *Classification of hypoxic-ischemic encephalopathy using long term heart rate variability based features* | Ahmed, R.; Temko, A.; Marnane, W. P.; Boylan, G.; Lightbody, G. | Study design |
| *Inflammatory cytokine response and reduced heart rate variability in newborns with hypoxic-ischemic encephalopathy* | Al-Shargabi, T.; Govindan, R. B.; Dave, R.; Metzler, M.; Wang, Y.; du Plessis, A.; Massaro, A. N. | Not published in journals with peer-review |
| *Diagnostic accuracy of fetal heart rate monitoring in the identification of neonatal encephalopathy* | Ater, S. B.; Murray, M. L.; Hunter, J. V. | Fetal population |
| *Heart rate variability in severely asphyxiated newborns during first few days of life correlates with long-term clinical outcome* | Bada, H. S.; Talati, A.; John, V.; Patwardhan, A. | Not published in journals with peer-review |
| *Correlation of heart rate variability with clinical outcome in severely asphyxiated newborns* | Bada, H.; Talati, A.; John, V.; Patwardhan, A.; Tse, H. F.; Lee, K. L.; Lau, C. P. | Not published in journals with peer-review |
| *Fetal asphyxia: prevention, detection and intervention* | Bertakis, K. D. | Fetal population |
| *Neonatal heart-rate-variability after intrauterine asphyxia and maternal obstretric regional analgesi* | Bratteby, L. E.; Andersson, L. | No grading of HIE |
| *Autonomic Dysfunction in Neonates with Hypoxic Ischemic Encephalopathy Undergoing Therapeutic Hypothermia Impairs Physiological Responses to Routine Care Events* | Campbell, H.; Govindan, R. B.; Kota, S.; Al-Shargabi, T.; Metzler, M.; Andescavage, N.; Chang, T.; Vezina, G.; du Plessis, A.; Massaro, A. N. | Irrelevant comparators |
| *[Recurrence plot analysis of HRV for brain ischemia and asphyxia]* | Chen, X.; Qiu, Y.; Zhu, Y. | Non-English language |
| *Heart rate changes are insensitive for detecting postasphyxial seizures in neonates* | Cherian, P. J.; Blok, J. H.; Swarte, R. M.; Govaert, P.; Visser, G. H. | No grading of HIE |
| *Diminished respiratory sinus arrhythmia in asphyxiated term infants* | Divon, M. Y.; Winkler, H.; Yeh, S. Y. | No grading of HIE |
| *Heart rate response to therapeutic hypothermia in infants with hypoxic-ischaemic encephalopathy* | Elstad, M.; Liu, X.; Thoresen, M. | Irrelevant comparators |
| *Automatic Detection of Artifact in Neonatal ECG* | Gholinezhadasnefestani, S.; Marnane, W.; Lightbody, G.; Temko, A.; Boylan, G.; Stevenson, N.; Ieee, | Not published in journals with peer-review |
| *Assessment of quality of ECG for accurate estimation of Heart Rate Variability in newborns* | Gholinezhadasnefestani, S.; Temko, A.; Stevenson, N.; Boylan, G.; Lightbody, G.; Marnane, W. | No grading of HIE |
| *Hypoxic-Ischemic Encephalopathy and Therapeutic Hypothermia: The Hemodynamic Perspective* | Giesinger, R. E.; Bailey, L. J.; Deshpande, P.; McNamara, P. J. | Study design |
| *Neonatal arrhythmias and indexes of heart rate variability in newborns* | Gonchar, M.; Ivanova, E.; Boichenko, A.; Kondratova, I.; Matsiyevska, N.; Teslenko, T. | Not published in journals with peer-review |
| *Heart rate variability in full-term neonates with hypoxic ischaemic encephalopathy* | Goulding, R. M.; Stevenson, N. J.; Murray, D. M.; Livingstone, V.; Boylan, G. B. | Not published in journals with peer-review |
| *Detrended fluctuation analysis of non-stationary cardiac beat-to-beat interval of sick infants* | Govindan, R. B.; Massaro, A. N.; Al-Shargabi, T.; Andescavage, N. N.; Chang, T.; Glass, P.; du Plessis, A. J. | No grading of HIE |
| *Neonatal heart rate reactivity following variable decelerations during labor* | Katz, M.; Sokal, M. M.; Lilling, M.; Fox, A. | Inclusion of healthy neonates only |
| *Changes of cardiovascular regulation during rewarming in newborns undergoing whole-body hypothermia* | Kozar, M.; Javorka, K.; Javorka, M.; Matasova, K.; Zibolen, M. | Not published in journals with peer-review |
| *The search continues: neuroprotection for all neonates with hypoxic-ischemic encephalopathy* | Lee, J. K.; Massaro, A. N.; Northington, F. J. | Study design |
| *It is hard to determine the relationship between heart rate variability and brain damage* | Li, W.; Wang, J.; Yao, Y. | Not published in journals with peer-review |
| *Autonomic responses in children with residual manifestations of early cerebral pathology* | Makarov, A. A.; Znamenskaya, E. I. | No grading of HIE |
| *Effect of temperature on heart rate variability in neonatal ICU patients with hypoxic-ischemic encephalopathy* | Massaro, A. N.; Campbell, H. E.; Metzler, M.; Al-Shargabi, T.; Wang, Y.; du Plessis, A.; Govindan, R. B. | Irrelevant comparators |
| *Heart rate variability in encephalopathic newborns during and after therapeutic hypothermia* | Massaro, A. N.; Govindan, R. B.; Al-Shargabi, T.; Andescavage, N. N.; Metzler, M.; Chang, T.; Glass, P.; du Plessis, A. J. | No grading of HIE |
| *Heart rate variability in newborns with hypoxic brain injury* | Matic, V.; Cherian, P. J.; Widjaja, D.; Jansen, K.; Naulaers, G.; Van Huffel, S.; De Vos, M. | Not published in journals with peer-review |
| *Pattern of brain injury and depressed heart rate variability in newborns with hypoxic ischemic encephalopathy* | Metzler, M.; Govindan, R.; Al-Shargabi, T.; Vezina, G.; Andescavage, N.; Wang, Y.; du Plessis, A.; Massaro, A. N. | No grading of HIE |
| *Heart rate variability in full-term normal and abnormal newborn infants during sleep* | Miyazaki, S.; Watanabe, K.; Hara, K. | No grading of HIE |
| *Clinical application of neonatal instantaneous heart rate monitoring (Part two: asphyxia and variability of instantaneous heart rate)* | Nishida, H.; Oguchi, K.; Haku, R.; Mihara, T.; Hiraishi, S.; Yashiro, K. | No grading of HIE |
| *Heart rate variability changes in neonatal hypoxic ischaemic encephalopathy* | O'Sullivan, P.; Korotchikova, I.; Doyle, O.; Murray, D.; Dempsey, E.; Boylan, G. | Not published in journals with peer-review |
| *Cardiorespirography in healthy and hypoxic newborn infants* | Pokorny, A.; Maly, Z.; Melkova, J.; Srp, B. | Non-English Language |
| *Continuous monitoring of heart rate variability in preterm infants* | Prietsch, V.; Knoepke, U.; Obladen, M. | No grading of HIE |
| *Analysis of the heart rate variability before and after asphyxia* | Rocha, F. C.; Schlindwein, F. S. | Not published in journals with peer-review |
| *Heart Rate Variability as a Marker for Asphyxia/Hypoxia* | Rocha, F. C.; Schlindwein, F. S.; Jan, J.; Kozumplik, J.; Provaznik, I. | Not published in journals with peer-review |
| *Effects of regional brain injury on the newborn autonomic nervous system* | Schneebaum Sender, N.; Govindan, R. B.; Sulemanji, M.; Al-Shargabi, T.; Lenin, R. B.; Eksioglu, Y. Z.; du Plessis, A. J. | No grading of HIE |
| *Cerebral modulation of the autonomic nervous system in term infants* | Schneebaum Sender, N.; Govindan, R. B.; Whitehead, M. T.; Massaro, A. N.; Metzler, M.; Wang, J.; Cheng, Y. I.; du Plessis, A. J. | No grading of HIE |
| *Cardiovascular changes during mild therapeutic hypothermia and rewarming in infants with hypoxic-ischemic encephalopathy* | Thoresen, M.; Whitelaw, A. | No HRV measurements |
| *The effect of therapeutic hypothermia on heart rate variability* | Vesoulis, Z. A.; Rao, R.; Trivedi, S. B.; Mathur, A. M. | Irrelevant comparators |
